# Causal relationships among perception of errors, challenges, and deliberate practice in athletes with disabilities

**DOI:** 10.3389/fpsyg.2024.1466848

**Published:** 2024-10-24

**Authors:** Young Kyun Sim, Joon Ha Shin, Song Eun Kim, Eun Chul Seo, Min-Seong Ha

**Affiliations:** ^1^Department of Physical Education, College of Sports Science, Woosuk University, Wanju-gun, Jeonbuk-do, Republic of Korea; ^2^Department of International Sports, College of Sports Science, Dankook University, Cheonan-si, Chungcheongnam-do, Republic of Korea; ^3^Department of Leisure and Recreation, College of Natural Sciences, Soonchunhyang University, Asan-si, Chungcheongnam-do, Republic of Korea; ^4^Department of Physical Education, College of Education, Wonkwang University, Iksan-si, Republic of Korea; ^5^Laboratory of Sports Conditioning, Nutrition Biochemistry and Neuroscience, Department of Sport Science, College of Arts and Sports, University of Seoul, Dongdaemun-gu, Seoul, Republic of Korea

**Keywords:** psychological conditioning, Taekwondo athlete, physical disability, hearing disability, structural equation model

## Abstract

**Introduction:**

There is limited evidence that the psychological characteristics of athletes with disabilities are identical to those of non-disabled athletes, owing to differences in ecological traits, and there is insufficient information on how athletes with disabilities perceive disabled athletes’ perception of errors, challenges, and deliberate practice. Therefore, it is necessary to examine whether the causal model of the perception of errors, challenge, and deliberate practice will be reproduced in the same way as in non-disabled athletes. Therefore, this study aimed to verify a causal model of the perception of errors, challenges, and deliberate practice by athletes with disabilities.

**Methods:**

The participants were 189 athletes with physical and hearing impairments (131 men and 58 women) registered with the 2023 Korea Paralympic Committee. Data were collected through a survey and the participants responded using a self-report method. The collected data were analyzed using descriptive statistics to verify normality, correlation analysis to examine relationships between variables, and structural equation modeling (SEM) to test the hypotheses.

**Results:**

Based on SEM analysis, the results of this study showed that the causal relationships between the perception of errors, challenges, and deliberate practice were partially significant. Specifically, perception of errors and reflection positively predicted challenges, whereas burden of mistakes negatively predicted challenges. Additionally, challenges were found to have a positive effect on deliberate practice.

**Discussion:**

By comprehensively examining the above, it can be interpreted as a major factor that can promote and reduce challenges depending on how athletes with disabilities perceive their mistakes.

## 1 Introduction

Errors experienced by athletes during training and competitions consistently occur as athletes attempt to reach their personal goals (Keith and Frese, 2008). Individual athletes perceive errors differently, which can affect their emotions and cognitive behaviors (Schell and Conte, 2008). As there is a close correlation between error recognition and athletic performance, these issues must be examined from various perspectives. The results of numerous studies on perception of error using multiple approaches have shown that perception of error has positive effects on athletic performance, along with numerous other factors. Lee et al. (2021) reported that those with a higher level of achievement goals tended to perceive errors more positively and take challenging approaches. Additionally, Sim et al. (2022) showed that a higher level of achievement goals positively affects the perception of errors and grit.

Research indicates that the perception of errors positively influences key psychological factors such as self-confidence, self-regulation, performance, grit, achievement goals, and perfectionism (Apró et al., 2024). These findings highlight the significance of adopting a constructive approach toward mistakes (Malureanu et al., 2021). Moreover, a positive perception of errors has been identified as a critical factor that enhances task-oriented goal pursuit and fosters the acquisition of new skills or solving complex problems, closely linking it to increased motivation (Farr et al., 1993). From a cognitive-behavioral perspective, focusing on errors shifts attention from outcome-based evaluations to a process-oriented mindset, providing individuals with opportunities for growth rather than withdrawal in the face of challenges. Previous research has predominantly aimed to predict variations in certain psychological constructs based on the perception of error. Challenges emerged as variables that may be significantly influenced by how individuals perceive mistakes.

Meanwhile, challenge refers to an individual’s disposition toward the perception of new and difficult tasks, which increases task performance and concentration (Hektner, 1997). It can also be defined as the positive desire to achieve personal goals (Csikszentmihalyi, 2011). This challenge was first introduced by the flow theory and is presented as a crucial factor that increases personal competence and athletic performance (Csikszentmihalyi, 2011). The conceptual trait of challenge plays an essential role in boosting athletes’ potential by triggering positive emotions and encouraging them to examine their weaknesses and train accordingly (Csikszentmihalyi, 2011). Challenge, in particular, has been shown to be closely associated with competitiveness, interest, effort, and practice (Sim et al., 2022). Previous studies on challenges have consistently suggested that they play a key role in explaining various traits related to personal achievement and success. Given its conceptual nature, challenge induces positive emotions and encourages individuals to reflect on their weaknesses and progressively improve, making it a critical factor in unlocking athletic potential (Csikszentmihalyi, 2011). As a result, a sense of challenge enhances deliberate practice.

Deliberate practice refers to the strategic and systematic behavior of individuals who understand their weaknesses, establish organized plans, and make endless efforts (Ericsson, 2009). It is a major determinant of athletes’ abilities because it is a critical element of professionalism and allows athletes to set advanced goals and take progressive measures even when encountering difficulties (Verner-Filion et al., 2017). As athletes need to use proper skills at the right time when performing competitions, setting persistent goals and maintaining professionalism through deliberate practice play critical roles.

Deliberative practices are closely associated with individuals’ motivations and attitudes. Duckworth et al. (2011) showed that deliberate practice compensates for athletes’ weaknesses and maximizes their strengths. As there are different perspectives on interpreting errors, the perception of errors reportedly affects one’s deliberate practice (Ford et al., 2009). In other words, to maintain self-directed deliberate practice, positive perception of errors and risk-taking attitudes are critical. Previous research on deliberate practice has focused on variables such as goal commitment, grit, satisfaction, self-regulation motivation, passion, and behavioral change, suggesting a close relationship between deliberate practice and individual motivation and attitude. Duckworth, Kirby, Tsukayama, Berstein, and Ericsson (Ericsson, 2009) posited that deliberate practice serves as a mechanism for athletes to compensate for their weaknesses and maximize their strengths. They also found that how athletes interpret mistakes influences deliberate practice, with the acceptance of mistakes playing a key role in this process (Fiori and Zuccheri, 2005; Ford et al., 2009). In other words, a positive perception of mistakes and a challenging attitude are crucial for sustaining self-directed deliberate practices.

Considering the points presented above, it can be predicted that error perception functions as a motivational factor for athletes, enhancing their sense of challenge, which, in turn, affects deliberate practice. However, error perception is a relatively recent motivational variable and its accumulation in subsequent research is limited. Although much research has been conducted in the field of education, its interpretation in the field of sports science is still ongoing. Additionally, most studies on deliberate practice have focused on elite athletes (Vink et al., 2015; Macnamara et al., 2016; Ericsson, 2020), but the accumulated evidence on consistent antecedent variables that explain deliberate practice remains limited.

Therefore, this study aimed to model the relationship between error perception and challenge as antecedent variables that can enhance deliberate practice in athletes, and examine the psychological characteristics that can improve athletic performance.

This study developed a model that could be applied to athletes with disabilities. There is limited evidence that the psychological characteristics of athletes with disabilities are the same as those of athletes without disabilities because of physiological differences (Sherrill, 1998). Therefore, little is known about the perception of errors, challenges, and deliberate practice of athletes with disabilities. Moreover, whether the causal model of the perception of errors, challenges, and deliberate practice can be applied to athletes with disabilities in the same way as those without disabilities needs to be evaluated. Hence, this study aimed to validate a causal model of the perception of errors, challenges, and deliberate practices in athletes with disabilities. Referring to the aforementioned studies, we developed the following hypothesis: Learning from errors, challenges, and the burden of mistakes will affect challenges. The perception of errors will affect challenges, which in turn will affect deliberate practice. A challenge will be a mediator between reflection of errors and deliberate practice.

## 2 Materials and methods

### 2.1 Participants

This study complied with the STROBE (Strengthening the Reporting of Observational Studies in Epidemiology) checklist, and the study protocol was approved by the Institutional Review Board at Wonkwang University, and was performed in compliance with the Helsinki Declaration and ethical research principles (WKIRB-202211-SB-111). The participants of this study were elite athletes with physical and hearing abilities registered with the 2023 Korea Paralympic Committee. The sample size of our study participants was calculated using G-power 3.1 (University of Kiel, Kiel, Germany). The estimated sample size was determined through an F-test for linear multiple regression, with [effect size: 0.15 (default), significance level: 0.05, power: 0.95], resulting in a total of 130 participants. Considering missing data, we recruited 207 participants. The purpose of the study was fully explained to the directors and authorities of the committee prior to the study, and 207 samples were collected through convenience sampling. A total of 189 participants were included in the final analysis after excluding 18 participants based on the following criteria: inconsistent responses across similar items, incomplete surveys with missing data, extreme or outlier values, non-random response patterns (e.g., selecting the same option throughout), and surveys completed in an unreasonably short or excessively long amount of time, indicating either a lack of attention or difficulty in understanding the questions. The exclusion criteria ensured the reliability and quality of the data used in the analysis. The sociodemographic characteristics of the participants are presented in Table 1.

**Table 1:** Characteristics of the participants.

Demographics	Category	Number of participants	N (%)
Gender	Male	131	69.3
	Female	58	30.6
Age	<20 years	48	25.4
	<30 years	77	40.7
	≥30 years	64	33.9
Career	Less than 2 years	37	19.6
	Less than 4 years	98	51.9
	Less than 4 years	27	15.8
	6 years or more	24	12.7
National competition award	Yes	112	59.3
	No	77	40.7
Total		189	100

### 2.2 Study instrument

To meet the aims of this research, the instrument used in this study was revised and adapted from a structured questionnaire used in previous studies. Particular attention was given to ensure that the items used in previous research could also be appropriately applied to athletes with disabilities and in the field of adaptive sports. To verify this, content validity was assessed by three experts in the field of adaptive sports (one professor of adapted physical education, one PhD in adapted physical education, and one coach specializing in disability sports), and all items were deemed acceptable for use. The questionnaire consisted of 34 questions: 4 items on sociodemographic characteristics, 17 items on perception of errors, 6 items on deliberate practice, and 7 items on challenge. The questionnaire content is presented in Table 2.

**Table 2:** Survey contents.

Categories	Contents	Items
Sociodemographic characteristics	Gender, age, experience, types of sports	4
Perception of errors	Learning from errors	4
	Challenging errors	4
	Burden of mistakes	4
	Reflection of errors	5
Challenge		7
Deliberate practice		6
Total		34

To examine the construct validity of the survey tool, confirmatory factor analysis (CFA) using the Maximum Likelihood (ML) estimation method and reliability analysis using Cronbach’s *α* were conducted. The model fit criteria were interpreted as follows: TLI and CFI values below 0.90, and SRMR and RMSEA values below 0.08 which considered to indicate good fit (Kline, 2023). Additionally, to assess convergent validity, the average variance extracted (AVE) and construct reliability (CR) were measured, with AVE values above 0.50 and CR values above 0.70 interpreted as indicating good fit (Fornell and Larcker, 1981; Anderson and Gerbing, 1988).

#### 2.2.1 Perception of errors

To measure the perception of errors, the Error Orientation Questionnaire (EOQ) developed by Rybowiak et al. (1999) and validated by Sim and Seo (2022) for Korean athletes was used. The adapted questionnaire consisted of 17 questions in four categories: 4 items for learning from errors, 4 items for challenging errors, 4 items for the burden of mistakes, and 5 items for reflecting on errors. The 5-point Likert Scale was used to analyze the responses.

#### 2.2.2 Challenge

To measure challenge, Student perceptions of classroom quality (SPOCQ), developed by Gentry and Owen (2004), translated by Lee and Choi (2016), and validated by Sim and Seo (2021) for Korean athletes were used in this study. The questionnaire consisted of six questions in a single category and a 5-point Likert Scale was used to analyze the responses.

#### 2.2.3 Deliberate practice

To measure deliberate practice, this study used a questionnaire developed by Vallerand et al. (2008), translated by Yang (2015), and validated by Sim and Seo (2020) for Korean athletes. The questionnaire consisted of five questions in a single category, and a 4-point Likert Scale was used to analyze the responses.

#### 2.2.4 Validity and reliability of the study instrument

To test the validity of the items in the aforementioned study instruments before their application, a team of experts (a professor of special physical education, an expert with a PhD in special physical education, and a sports manager working with disabled athletes) validated the questionnaires. Furthermore, to verify the evidence for construct validity, a confirmatory factor analysis (CFA) based on maximum likelihood (ML) estimation and reliability analysis using Cronbach’s *α* was performed. Here, the standards for the goodness of fit of the model were set as TLI and CFA ≤0.90, and the SRMR and RMSEA ≤0.80 (Kline, 2023). To examine convergent validity, average variance extracted (AVE) and construct reliability (CR) were measured. The standards for AVE was ≥0.50 and CR was ≥0.70 for good fit (Fornell and Larcker, 1981; Anderson and Gerbing, 1988).

#### 2.2.5 Confirmatory factor analysis and reliability of perception of errors

Confirmatory analysis of perception of errors showed a fitness of *χ*^2^ = 177, df = 113, TLI = 0.958, CFI = 0.966, SRMR = 0.052, and RMSEA = 0.054. The reliability was 0.916 for learning from errors, 0.928 for challenging errors, 0.843 for the burden of mistakes, and 0.891 for the reflection of errors (Table 3). Hence, the scale for perception of errors used in this study satisfied the evidence of construct validity.

**Table 3:** Confirmatory factor analysis and reliability analysis of perception of errors, challenge, deliberate practice.

Latent variable		Variable	*B*	*β*	S.E	*t*	AVE	C.R	*α*
Perception of errors	Learning from errors	a1	0.777	0.851	0.054	14.28***	0.930	0.981	0.916
		a2	0.791	0.878	0.052	15.00***			
		a3	0.872	0.896	0.056	15.51***			
		a4	0.779	0.803	0.059	13.03***			
	Challenging errors	a5	0.567	0.664	0.059	9.55***	0.895	0.971	0.829
		a6	0.785	0.77	0.067	11.67***			
		a7	0.734	0.76	0.063	11.48***			
		a8	0.826	0.779	0.069	11.87***			
	Burden of mistakes	a9	0.730	0.694	0.07	10.40***	0.898	0.972	0.843
		a10	0.989	0.916	0.065	15.20***			
		a11	0.814	0.779	0.067	12.07***			
		a12	0.601	0.645	0.063	9.46***			
	Reflection of errors	a13	0.587	0.712	0.054	10.86***	0.921	0.983	0.891
		a14	0.688	0.852	0.048	14.08***			
		a15	0.641	0.756	0.054	11.82***			
		a16	0.713	0.836	0.052	13.70***			
		a17	0.714	0.788	0.057	12.49***			
*χ*^2^ = 177, df = 113, TLI = 0.958, CFI = 0.966, SRMR = 0.052, RMSEA = 0.054									
Challenge		b1	0.752	0.833	0.054	12.9***	0.925	0.987	0.923
		b2	0.794	0.872	0.053	15.5***			
		b3	0.780	0.887	0.05	12.5***			
		b4	0.688	0.787	0.054	14.5***			
		b5	0.761	0.833	0.054	10.7***			
		b6	0.618	0.691	0.058	10.7***			
*χ*^2^ = 13.3, df = 9, TLI = 0.991, CFI = 0.995, SRMR = 0.015, RMSEA = 0.050									
Deliberate practice		c1	0.490	0.612	0.056	8.70***	0.905	0.979	0.844
		c2	0.609	0.728	0.055	10.92***			
		c3	0.614	0.76	0.052	11.65***			
		c4	0.757	0.867	0.054	13.98***			
		c5	0.523	0.629	0.058	9.02***			
*χ*^2^ = 12.3, df = 5, TLI = 0.960, CFI = 0.980, SRMR = 0.028, RMSEA = 0.077									

***p < 0.001.

#### 2.2.6 Confirmatory factor analysis and reliability of challenge

Confirmatory analysis of challenge showed the fitness of *χ*^2^ = 13.3, df = 9, TLI = 0.991, CFI = 0.995, SRMR = 0.015, and RMSEA = 0.050. Reliability was 0.923 for challenge (Table 3). Hence, the scale for challenge used in this study provides evidence of construct validity.

#### 2.2.7 Confirmatory factor analysis and reliability of deliberate practice

Confirmatory analysis of challenge showed the fitness of *χ*^2^ = 12.3, df = 5, TLI = 0.960, CFI = 0.980, SRMR = 0.028, and RMSEA = 0.077. Reliability was 0.844 for deliberate practice (Table 3). Hence, the scale for deliberate practice used in this study demonstrated construct validity.

### 2.3 Procedure

Data were collected via face-to-face surveys. The researchers contacted the representatives or team officials of the athletes’ organizations in advance to recruit participants. Visits were scheduled only for teams that agreed to cooperate and provided approval for the study. The research team then visited the teams according to their schedules. Participants were provided with detailed explanations of the study’s purpose, methods, and ethical considerations. It was emphasized that participation should not be influenced by coercion from coaches or team officials and that there would be no disadvantages for those who chose not to participate. For teams that included athletes with hearing disabilities, information was conveyed through sign language with the assistance of a specialist. Subsequently, the participants signed consent forms, and the surveys were distributed. The participants completed the surveys using a self-report method and the completed surveys were collected immediately. The collected surveys underwent coding and data cleaning, and were then analyzed according to the study’s purpose and methods.

### 2.4 Data analysis

Processing of data collected from this study were performed using Jamovi 2.0 (IBM, New York, USA) and AMOS 23.0 (IBM, New York, USA) to validate the hypothesis. Significance level (*α*) was set as 0.05. A detailed analysis is provided below.

A frequency analysis was conducted for sociodemographic characteristics. Confirmatory factor analysis (CFA) based on maximum likelihood (ML) estimation and reliability analysis were conducted to verify the evidence for construct validity. In addition, skewness and kurtosis were analyzed to test normality, and Pearson’s R correlation analysis were performed to examine the relationships between major variables. Finally, prior to validating the study model, the goodness of fit for measurement models proposed by Anderson and Gerbing (1988) was reviewed, and the structural model was analyzed.

## 3 Results

### 3.1 Normality test

As the estimation of the measurement and structural models was based on maximum likelihood (ML) in this study, normality was tested, which is its underlying assumption. The results showed the skewness of −0.424 ~ 0.389 and kurtosis of −0.612 ~ 0.543 as shown in Table 4, which met the standards proposed by Kline (2023) (skewness ≤ ±3, kurtosis ≤ ±8).

**Table 4:** Normality test.

		Skewness		Kurtosis	
		S	SEM	S	SEM
Perception of errors	Learning	−0.424	0.177	0.543	0.352
	Challenge	−0.003		−0.612	
	Strain	−0.244		0.096	
	Reflection	0.389		−0.464	
Challenge		−0.227		−0.15	
Deliberate practice		0.254		−0.242	

### 3.2 Correlation analysis

Pearson’s R correlation analysis was conducted to examine the relationships among the perception of errors, challenges, and deliberate practice. As shown in Table 5, the sub-variables of the three variables (perception of errors, challenges, and deliberate practice) had partial correlations. All coefficients were ≤ 0.80, which is the standard of multicollinearity, indicating that the concepts of the three variables (perception of errors, challenge, and deliberate practice) did not overlap (Kline, 2023).

**Table 5:** Pearson’s r correlation analysis of perception of errors, challenge, and deliberate practice.

	1	2	3	4	5	6
Learning from errors	1					
Challenging errors	0.570**	1				
Burden of mistakes	0.085	0.019	1			
Reflection of errors	0.397**	0.412**	0.375**	1		
Challenge	0.408**	0.471**	−0.083	0.390**	1	
Deliberate practice	0.362**	0.388**	0.077	0.574**	0.633**	1

**p < 0.01.

### 3.3 Validation of the measurement model

The measurement model was first validated before using the structural equation model (SEM) according to Anderson and Gerbing (1988). The pathway for validating the SEM was saturated, and the goodness-of-fit of the measurement model was tested. The result demonstrated that the goodness of fit described by *χ*^2^ = 545.528, df = 0.339, TLI = 0.930, CFI = 0.938, SRMR = 0.075, and RMSEA = 0.057 met the standards (Kline, 2023). Moreover, the standardized coefficients (*β*) of each latent variables explaining measurement variables were 0.648 ∼ 908 for perception of errors, 0.691 ∼ 829 for challenge, and 0.605 ∼ 851 for deliberate practice. As this validated the explanatory power of the measurement variables, SEM was analyzed. The details of measurement model validation are shown below (Table 6).

**Table 6:** Measurement model validation.

Latent variables	Measurement variables	*B*	*β*	*t*
Learning from errors	a1	1	0.853	Criterion variable
	a2	1.016	0.877	15.536***
	a3	1.121	0.895	16.074***
	a4	1.002	0.803	13.454***
Challenging errors	a5	1	0.661	Criterion variable
	a6	1.384	0.766	8.699***
	a7	1.317	0.77	8.726***
	a8	1.453	0.774	8.761***
Burden of mistakes	a9	1	0.648	Criterion variable
	a10	1.623	0.908	9.655***
	a11	1.361	0.786	8.999***
	a12	1.213	0.696	8.177***
Reflection of errors	a13	1	0.707	Criterion variable
	a14	1.177	0.849	10.843***
	a15	1.109	0.763	9.815***
	a16	1.224	0.837	10.702***
	a17	1.226	0.788	10.124***
Challenge	b1	1	0.829	Criterion variable
	b2	1.064	0.874	14.944***
	b3	1.041	0.887	15.313***
	b4	0.917	0.785	12.668***
	b5	1.016	0.833	13.851***
	b6	0.826	0.691	13.851***
Deliberate practice	c1	1	0.605	Criterion variable
	c2	1.203	0.696	7.596***
	c3	1.316	0.789	8.266***
	c4	1.534	0.851	8.625***
	c5	1.126	0.656	7.280***
*χ*^2^ = 545.528, df = 0.339, TLI = 0.930, CFI = 0.938, SRMR = 0.075, RMSEA = 0.057				

***p < 0.001.

### 3.4 Validation of the structural model

To statistically determine whether the hypothesis was accepted or rejected. The perception of errors of elite athletes with disabilities (learning from errors, challenging errors, burden of mistakes, and reflection or errors) was set as an exogenous and independent variable, and challenge was set as an endogenous and mediating variable. Deliberate practices were used as the dependent variables. The goodness of fit of the model described by *χ*^2^ = 545.528, df = 0.339, TLI = 0.930, CFI = 0.938, SRMR = 0.075, and RMSEA = 0.057 met the standards proposed by Kline (2023) (Table 7). The details of hypothesis testing are presented below.

**Table 7:** Goodness of fit of the model.

Latent variables			*B*	*β*	S.E	*t*	Hypothesis testing
Hypothesis 1	Learning from errors	Challenge	0.147	0.152	0.088	1.666	Reject
Hypothesis 2	Challenging errors		0.324	0.244	0.139	2.333***	Accept
Hypothesis 3	Burden of mistakes		−0.36	−0.291	0.101	−3.580***	Accept
Hypothesis 4	Reflection of errors		0.521	0.406	0.122	4.275***	Accept
Hypothesis 5	Challenge	Deliberate practice	0.471	0.727	0.065	7.221***	Accept
Goodness of fit: *χ*^2^ = 545.528, df = 0.339, TLI = 0.930, CFI = 0.938, SRMR = 0.075, RMSEA = 0.057							

***p < 0.001.

*H1*: Learning from errors did not have a statistically significant effect on challenge.

*H2*: Challenging errors had a positive (+) effect on challenge (*β* = 0.244, *t* = 2.333***).

*H3*: Burden of mistakes had a negative (−) effect on challenge (*β* = −0.291, *t* = −3.580***).

*H4*: Reflection of errors had a positive (+) effect on challenge (*β* = 0.406, *t* = 4.275***).

*H5*: Challenge had a positive (+) effect on deliberate practice (*β* = 0.727, *t* = 7.221***).

The overall analysis of the results showed that challenging errors and reflection of errors, the sub-variables of perception of errors, had positive (+) effects on challenges, whereas the burden of mistakes had a negative (−) effect. When the perception of errors was controlled, challenge had a positive (+) effect on deliberate practice. In other words, an increase in challenging errors and reflection of errors leads to an increase in challenges, ultimately promoting deliberate practice. On the contrary, an increase in the burden of mistakes leads to a decrease in challenges, discouraging deliberate practice. Therefore, perception of errors (challenging errors, mistakes, and errors) can explain deliberate practice through the mediation of challenges.

To determine whether the effects of the pathways from these results were statistically significant, the indirect effects (mediating effects) of these variables were validated for statistical significance (Baron and Kenny, 1986). To test the indirect effect (mediating effect) between the perception of error (challenging errors, burden of mistakes, and reflection of errors) and deliberate practice, the bootstrap method was conducted with 2,000 replications, and statistical significance was determined at a bias-corrected 95% confidence interval (Shrout and Bolger, 2002). A detailed validation of the statistical significance of the mediating effects is presented in Table 8.

**Table 8:** Statistical significance of the mediating effect.

Latent variables			Lower bounds	Upper bounds	Indirect effects	*p*	Testing result
Challenging errors	Challenge	Deliberate practice	−0.008	0.367	0.172	0.063	Reject
Burden of mistakes	Challenge	Deliberate practice	−0.321	−0.084	−0.191	0.001	Accept
Reflection of errors	Challenge	Deliberate practice	0.121	0.471	0.276	0.001	Accept

***p < 0.001.

The mediating effect of challenge on the relationship between challenging errors and deliberate practice was not statistically significant. In the relationship between burden of mistakes and deliberate practice, the lower and upper bound values of challenge did not include ‘0,’ which indicates a statistical significance of the mediating effect (Shrout and Bolger, 2002). This suggests that an increase in the burden of mistakes leads to a decrease in challenges, ultimately reducing deliberate practice. In the relationship between reflection of errors and deliberate practice, the lower and upper bound values of challenge did not include ‘0,’ which indicates a statistical significance of the mediating effect (Shrout and Bolger, 2002). This suggests that an increase in reflection of errors leads to an increase in challenge and ultimately promotes deliberate practice, which supports hypothesis 4.

## 4 Discussion

This study aimed to elucidate the relationships among perception of errors, challenges, and deliberate practices among elite athletes with disabilities. The structural equation was analyzed using the sub-variables of perception of errors – learning from errors, challenging errors, burden of mistakes, and reflection of errors–as independent variables, challenge as a mediating variable, and deliberate practice as a dependent variable. A discussion of the results based on this setting is as follows.

The analysis of the effect of the perception of errors (learning from errors, challenging errors, burden of mistakes, and reflection of errors) on challenge showed that the perception of errors partially affected the challenge. The detailed results are discussed below.

First, learning from errors did not have a statistically significant effect on challenge. This does not support the results of previous studies, which reported that various learning experiences enhance the behavior of challenge (Bird, 2004; Starbuck and Farjoun, 2005; Bauer, 2008; Roberts and Treasure, 2012). However, our results can be explained from the perspective of the decomposition of effects (Kline, 2023). Learning from errors refers to utilizing information obtained from errors. The fact that learning from errors did not affect challenge indicates that the roles of reflection and challenge rather than learning itself, have a greater effect on increasing challenges. In other words, the reflection of errors and challenging errors had strong effects on challenge among the total effect of the structural model, and it is likely that these effects were decomposed from the total effect. Correlation analysis showed a positive and statistically significant correlation between learning from errors and challenges. Therefore, learning from errors is likely to affect challenge. Roberts (Roberts and Treasure, 2012) reported that meeting the desire for challenge through continuous learning is crucial for improving athletic performance. Vaughan (Starbuck and Farjoun, 2005) showed that it also reduced negative patterns and slumps. These perspectives suggest that learning from errors enhances athletes’ performance, underscoring the importance of a positive view on learning from errors. Providing stepwise goals to athletes with disabilities and reducing negative elements through regular counseling will be needed to establish positive psychological well-being in these athletes. Furthermore, efforts must be made to promote athletes’ ability to learn from errors by monitoring videos of training or competitions and providing useful feedback and information.

Second, challenging errors were found to have a positive (+) effect on challenges. This result supports previous studies that reported the importance of risk-taking behavior in increasing the desire for challenges (Starbuck and Farjoun, 2005; Csikszentmihalyi, 2011; Lim and Yoon, 2017). Farr et al. (1993) suggest that cultivating a behavioral desire toward a challenge is important for decreasing the frequency of errors. Csikszentmihalyi (2011) proposed that an adventurous attitude toward personal growth acts as a mechanism that promotes challenges. In other words, challenging errors are a positive phenomenon that changes athletes’ behavior and yields better outcomes.

The challenging attitude toward errors, characterized by behavioral tendencies, is reported to be critical for enhancing athletes’ static skills, and their technique and concentration levels (Csikszentmihalyi, 2011; Dweck et al., 2014). Therefore, managers should encourage athletes’ positive self-efficacy so that they do not fear error. Since self-efficacy is determined by the experience of success, verbal persuasion, and physical-psychological state (Kyun and EunChul, 2020), managers should explore various management strategies promoting behavioral characteristics of athletes with disabilities to enhance their self-efficacy and challenging attitude toward errors.

Third, the burden of mistakes has a negative (−) effect on challenges. This result supports numerous previous studies reporting that the fear of making errors reduces the desire for challenge (Bandura et al., 1999). Van Dyck et al. (2005) showed that the burden of mistakes leads to avoidance of challenges and negative emotions in athletes, which reduces their individual skills and performance. Hence, the burden of mistakes is likely to decrease athletes’ motivational desire, hindering their performance capabilities (Keith and Frese, 2008; Schwebel et al., 2016). Managers must be aware that setting an atmosphere of overly focusing on winning can increase the burden of mistakes on athletes (Sherrill, 1998). They need to make efforts to emphasize the process and not the outcome to reduce the burden of mistakes. Furthermore, athletes with disabilities should develop positive emotions through a consistent reputation and image training to reduce the burden of mistakes.

Fourth, reflection of errors had a positive (+) effect on challenges. This result supports the theory that reflection on one’s errors and a strong will to avoid repeating the same errors are critical in pursuing continuous challenges (Sherrill, 1998; Roberts and Treasure, 2012). Bauer (2008) proposed that boosting intrinsic motivation (e.g., challenge, achievement, and interest) is important for reflecting on errors and achieving one’s goals. Reflection of errors can be strengthened by informational feedback, which is specific and useful information conveyed by managers to athletes about their errors (Sherrill, 1998). Hence, managers can improve athletes’ challenges by providing consistent feedback through various strategies, such as recording training, logging performance, and providing peer review.

Our analysis of the effects of challenge on deliberate practice in elite athletes with disabilities showed that challenges positively (+) effect on deliberate practice. This supports previous findings that the desire for challenge promotes deliberate practice (Starkes and Ericsson, 2003; Yang, 2015). Starkes and Ericsson (2003) state that developing a positive desire for challenge is essential for facilitating continuous deliberate practice. This is consistent with Ericsson’s study (Starkes and Ericsson, 2003), which proposed that challenges should precede systematic and strategic practice. Challenge is an essential desire of athletes to encourage professionalism and promote positive outcomes.

Validation of the mediating effect of challenge on the relationship between perception of errors and deliberate practice in elite athletes with disabilities showed that challenge did not have a statistically significant effect on the relationship between challenging errors and deliberate practice. However, challenge had a statistically significant mediating effect on the relationship between the burden of mistakes, reflection of errors, and deliberate practice. These results show that the burden of mistakes and reflection of errors explain deliberate practice through challenge; notably, taking into account the strongest effect of reflection of errors, athletes’ willingness to actively reflect on their errors and avoid making the same errors will be most critical in promoting deliberate practice in athletes with disabilities (Starkes and Ericsson, 2003; Ericsson, 2009). Therefore, managers should make constant efforts to encourage athletes to reflect on their errors to enhance their deliberate practice. This will equip them with coaching strategies that can better manage the errors made by athletes with disabilities, which is a natural phenomenon that will ultimately improve their athletic performance.

## 5 Conclusion

This study elucidated the relationship among the perception of errors, challenges, and deliberate practices in athletes with disabilities. The results are as follows: First, learning from errors did not have a statistically significant effect on challenge. Second, challenging errors had a positive (+) effect on challenges. Third, the burden of mistakes has a negative (−) effect on challenges. Fourth, reflection of errors had a positive (+) effect on challenges. Fifth, challenge had a positive (+) effect on deliberate practice; finally, the mediating effect of challenge did not have a statistically significant effect on the relationship between challenging errors and deliberate practice. However, challenge had a negative (−) effect on the relationship between the burden of mistakes and deliberate practice and a positive (+) effect on the relationship between reflection of errors and deliberate practice with statistical significance.

Overall, the perception of errors in athletes with disabilities is a major factor that can facilitate or discourage challenges. In other words, our study suggests that the way athletes with disabilities perceive their errors can be an antecedent variable that can alter their challenges and actual performance (deliberate practice).

## 6 Limitation and future directions

Our study had several limitations. First, although the participants in our study included athletes with physical and hearing disabilities, we did not control for differences between these two groups. The results may vary depending on the severity or type of the disability. Therefore, future research should classify athletes with disabilities into separate groups and conduct multigroup analyses to verify the differences in the research model. Second, because our study focused on Taekwondo athletes with disabilities, there are limitations in applying the findings to non-disabled populations. Third, we concentrated on the variables related to the perception of errors, challenges, and deliberate practice. However, further efforts are required to strengthen and generalize our research model. For example, a more detailed analysis is required to understand the role of challenges (e.g., mediating or moderating effects) in the relationship between perception errors and deliberate practice among athletes with disabilities. Additionally, expanding the model by incorporating variables related to performance is expected to provide further insights. Fourth, there are limitations to the measurement tools. The tools used in this study were adapted for athletes with disabilities, using tools originally designed for non-disabled athletes. Although there were no issues with content validity, the measurement items were not specifically developed for athletes with disabilities and thus may not fully capture the constructs. Therefore, future research should focus on developing items to assess the perception of errors, challenges, and deliberate practices, particularly for athletes with disabilities.

## Data Availability

The original contributions presented in the study are included in the article/supplementary material, further inquiries can be directed to the corresponding authors.
